# Releasing stimuli and aggression in crickets: octopamine promotes escalation and maintenance but not initiation

**DOI:** 10.3389/fnbeh.2015.00095

**Published:** 2015-04-21

**Authors:** Jan Rillich, Paul A. Stevenson

**Affiliations:** ^1^Institute for Neurobiology, Free University of BerlinBerlin, Germany; ^2^Institute for Biology, Leipzig UniversityLeipzig, Germany

**Keywords:** animal conflict, dopamine, serotonin, behavioral pharmacology, priming, social status, neuromodulation, winner effect

## Abstract

Biogenic amines have widespread effects on numerous behaviors, but their natural functions are often unclear. We investigated the role of octopamine (OA), the invertebrate analog of noradrenaline, on initiation and maintenance of aggression in male crickets of different social status. The key-releasing stimulus for aggression is antennal fencing between males, a behavior occurring naturally on initial contact. We show that mechanical antennal stimulation (AS) alone is sufficient to initiate an aggressive response (mandible threat display). The efficacy of AS as an aggression releasing stimulus was augmented in winners of a previous fight, but unaffected in losers. The efficacy of AS was not, however, influenced by OA receptor (OAR) agonists or antagonists, regardless of social status. Additional experiments indicate that the efficacy of AS is also not influenced by dopamine (DA) or serotonin (5HT). In addition to initiating an aggressive response, prior AS enhanced aggression exhibited in subsequent fights, whereby AS with a male antenna was now necessary, indicating a role for male contact pheromones. This priming effect of male-AS on subsequent aggression was dependent on OA since it was blocked by OAR-antagonists, and enhanced by OAR-agonists. Together our data reveal that neither OA, DA nor 5HT are required for initiating aggression in crickets, nor do these amines influence the efficacy of the natural releasing stimulus to initiate aggression. OA's natural function is restricted to promoting escalation and maintenance of aggression once initiated, and this can be invoked by numerous experiences, including prior contact with a male antenna as shown here.

## Introduction

Biogenic amines are renown for their role in orchestrating and generating numerous aspects of animal behavior. Notably, the invertebrate analog of noradrenaline, octopamine (OA), is reputed to play key roles in initiating and modulating simple patterns of locomotion and controlling complex social interactions (reviews: Verlinden et al., 2010; Simpson and Stevenson, 2014). Our work on male crickets was the first to show that OA promotes the expression of intra-specific aggression in an invertebrate (Stevenson et al., 2000, 2005), rather than serotonin (5HT) as found in crustaceans (review: Kravitz and Huber, 2003), and this has now been confirmed for various other insects (e.g., fruit flies: Baier et al., 2002; Hoyer et al., 2008; Zhou et al., 2008; ants: Aonuma and Watanabe, 2012; stalk-eyed flies: Bubak et al., 2014). Crickets exhibit spectacular fighting behavior (Alexander, 1961) and, as in mammals (Nelson, 2006), their aggressiveness is influenced by a wide variety of social and other experiences (e.g., Adamo and Hoy, 1995; Killian and Allen, 2008), and this is where OA plays a key role. We have shown that experiences as diverse as physical exertion (Stevenson et al., 2005), winning a fight (Rillich and Stevenson, 2011), and resource possession (Rillich et al., 2011) each promote the expression of aggressive behavior *via* the activation of the octopaminergic system (reviews: Stevenson and Rillich, 2012; Stevenson and Schildberger, 2013; Simpson and Stevenson, 2014).

In this paper we address the potential role of OA in actually initiating aggressive behavior. OA has long been known to activate central pattern generators such as that underlying locust flight (Stevenson and Kutsch, 1988), although recent studies revealed that OA acts rather to augment the efficacy of cholinergic neurons activated by the natural flight-releasing stimulus (Buhl et al., 2008). The key-releasing stimulus for aggression in crickets is antennal contact between conspecific males, which occurs naturally during antennal fencing, a behavior executed immediately when they first meet (Hofmann and Schildberger, 2001).

The antenna of crickets, and other insects, is a complex multimodal sensory organ, equipped with various and numerous mechanoreceptors and olfactory receptors (review: Staudacher et al., 2005), and signals from both types are each thought to be important in controlling the decision to fight or court a conspecific in crickets (Rence and Loher, 1977; Hofmann and Schildberger, 2001; Nagamoto et al., 2005; Iwasaki and Katagiri, 2008; Sakura and Aonuma, 2013) and fruit flies (Vrontou et al., 2006; Certel et al., 2007; Chan and Kravitz, 2007; Wang and Anderson, 2010; Andrews et al., 2014).

Insect antennae also contain release sites for OA (Pass et al., 1988; Allgäuer and Honegger, 1993). Amines are known to directly modulate both mechano- (Ramirez et al., 1993) and pheromone receptor cells in the periphery (Pophof, 2000; Schendzielorz et al., 2015), as well as synaptic transmission between these afferents and follower cells in the insect central nervous system (e.g., Kloppenburg et al., 1999; Leitch et al., 2003). Antennal stimulation (AS) has also been shown to excite octopaminergic neurons, at least in locusts (Duch et al., 1999) and lead to elevated levels of OA in the heamolymph of crickets (Adamo et al., 1995), but the consequences of this for the expression of aggression is not known. This study addresses explicitly whether OA initiates or modulates aggressive behavior. We describe two effects of AS on aggression, an immediate initiating effect, and a priming effect on subsequent fighting behavior. We go on to evaluate the extent to which each is modulated by amines using established drugs. Our results show that OA's natural function is restricted to promoting escalation and maintenance of aggression once it has been initiated, but is not required for initiation itself.

## Material and methods

### Experimental animals

Mature, 2–3 week old, adult male Mediterranean field crickets, *Gryllus bimaculatus* (de Geer) were taken from a breeding stock maintained under constant standard conditions at Leipzig University (22–24°C, relative humidity 40–60%, 12 h: 12 h light: dark regime daily feeding on bran and fresh vegetables). All experiments were performed during daylight hours, avoiding times when aggression tends to be depressed (just after midday and on generally dreary days; cf. Stevenson et al., 2000). All animal treatments complied with the Principles of Laboratory Animal Care and the German Law on the Protection of Animals (Deutsches Tierschutzgesetz). Our investigation is based on analysis of crickets that had the following social experiences:

#### Socially naïve

These crickets were kept isolated in individual glass jars for 18–24 h prior to all experiments, after which all known effects of previous social interactions on aggressive behavior have abated (Stevenson and Rillich, 2013).

#### Losers and winners

Losers and winners were crickets that had clearly lost or won a fight. They were established by matching social naive contestants. The losers are the first to retreat and typically avoid conspecific males for 1–3 h after defeat (“loser effect”—Stevenson and Rillich, 2013), while winners become highly aggressive and typically generate the rival song and body jerks (“winner effect”—Rillich and Stevenson, 2011). The hierarchical relationship was verified <1 min after the initial fight to assure that the designated loser retreated immediately from the designated winner. Experiments with winners and losers were performed 15 min after the initial fight.

### Evaluation of aggression

Aggressive behavior was evaluated in dyadic contests between equally sized males (<5% weight difference) and thus equal win chances (Rillich et al., 2007). The opponents were placed at opposite ends of a clear Perspex-glass rectangular fighting arena (l. w. h.: 16 × 9 × 7 cm) with a sand-covered floor divided halfway along its length by an opaque sliding door. On removing the door the animals' interactions follow a stereotyped sequence typical for fights in the field (Alexander, 1961) which we score on a scale of 0–6 to denote aggressive escalation (Hofmann and Stevenson, 2000; Stevenson et al., 2000): Level 0: mutual avoidance without aggression. Level 1: one cricket attacks, the other retreats. Level 2: antennal fencing. Level 3: mandible spreading by one cricket. Level 4: mandible spreading by both crickets. Level 5: mandible engagement. Level 6: grappling, an all-out fight. Contests can finish at any level with the retreat of one opponent, and fight duration was measured to the nearest second with a stopwatch, deducting pauses that occasionally occurred when the animals lost contact.

### Antennal stimulation

Freshly excised antennae were obtained from mature, adult male and female donor crickets, some of which were washed twice for 10 min with n-hexane to remove cuticular pheromones (Iwasaki and Katagiri, 2008). Test crickets were stimulated with donor antenna by stroking their antennae continually for 30 s. Behavioral responses to AS were noted during stimulation, and its influence on fighting behavior was analyzed 5 min later.

### Pharmacological treatments

The role of biogenic amines in controlling initiation of aggression was tested by applying the following aminergic drugs, which were obtained from Sigma-Aldrich (Deisenhofen, Germany): The tissue permeable octopamine receptor (OAR) agonist chlordimeform hydrochloride (CDM, cf. Roeder, 1995), the dopamine receptor (DAR) agonist homovanillyl alcohol (HVA: Beggs and Mercer, 2009; Rillich and Stevenson, 2014), the selective OAR-blocker epinastine hydrochloride (Roeder et al., 1998), the insect DAR-blocker fluphenazine dihydrochloride (Degen et al., 2000) and the competitive serotonin (5HT) synthesis inhibitor alpha-methyltryptophan (AMTP). The amine receptor agonists and antagonists were dissolved in 1% dimethylsulfoxide (DMSO) in insect saline (constituents in mM: NaCl 140, KCl 10, CaCl_2_ 7, NaHCO_3_ 8, MgCl_2_ 1, N-trismethyl-2-aminoethanesulfonic acid 5, d-trehalose dihydrate, pH 7.4) and the 5HT synthesis blocker AMTP in saline alone. All drugs were injecting into the hemocoel *via* the pronotum using a micro-syringe (Hamiliton®, Bonaduz, Switzerland). The most effective concentrations and time span for inducing noticeable changes in cricket aggressive behavior, without any obvious detrimental effect on general motility have been determined in previous studies (Stevenson et al., 2000; Rillich and Stevenson, 2014). Accordingly, each cricket treated with an aminergic agonist received a single 10 μl dose of 1 mM CDM or HVA, those that received antagonist a single 10 μl dose of 20 mM epinastine or fluphenazine, and those that received the 5HT synthesis blocker 3 successive injections of 1.0 mg AMTP in 40 μl saline administered at 48 h intervals. Crickets treated with vehicle alone served as controls. The effects of the amine receptor agonists and antagonists on the response to AS and on fighting behavior were tested 30–60 min after their application. This minimized effects of handling prior to AS, and allowed sufficient time for the drugs to be effective (cf. Rillich and Stevenson, 2014). Animals treated with AMTP were tested 48 h after the last injection, at which time 5HT levels have been shown to be below the detection levels of HPLC (Sloley and Orikasa, 1988). Furthermore, to minimize variability due to random day-to-day differences in performance, we took the precaution of evaluating single pairs of crickets from vehicle-treated and drug-treated groups in parallel and accumulated data from successive daily experiments (maximum three different treatments per day).

### Antennectomy and 5HT immunocytochemistry

Both antennae of young adult male crickets were cut off at the pedicellae and the animals sacrificed 7 days later. Their brains were then dissected out, and 5HT immunocytochemistry performed using an established antiserum as described in detail elsewhere (Stevenson et al., 2000). Briefly, brains were fixed (2 h, 2.5% paraformaldehyde in phosphate buffered saline, PBS), dehydrated by passing through an ascending ethanol series, cleared in xylene and embedded in paraffin wax (1 h, 58°C). Horizontal sections (14 μm, relative to embryonic axis) were cut with a microtome (Jung-Biocut, Leica, Wetzlar, Germany) and mounted on glass slides. Slides were then passed through xylene, rehydrated in a descending alcohol series and bathed in PBS containing 0.1% Triton-X100 (PBS-Tx) followed by incubated in normal goat serum (NGS, Sigma, 1 h, 10% in PBS-Tx) and then serotonin antiserum (rabbit, polyclonal, Eugene Tech, Inc., Ridgefield Park, NJ, USA; 1:1500 in PBS-Tx, 1% NGS, overnight). After washing, slides were incubated in Alexa 488-tagged goat anti-rabbit serum (Invitrogen, Carlsbad, CA, USA, 1:500, 2 h), washed, dehydrated, cleared in xylene and mounted in Entellan (Merck, Darmstadt, Germany) under coverslips. Sections were viewed and scanned with a confocal microscope (TCS STED, Leica Microsystems, Wetzlar, Germany). Image colors were inverted and then converted to black and white using Canvas-X (ACD-Systems), but not otherwise manipulated.

### Data analysis

All statistical tests were performed in Prism 5 (GraphPad Software Inc., La Jolla, CA, USA) running on a Macintosh computer (Apple Computers, Cupertino, CA, USA). The median and the interquartile range (IQR) were calculated for non-parametric data sets. Non-parametric tests were also performed on duration since the data sets failed D'Agostino and Pearson omnibus normality tests, even after log transformations. The χ^2^ test was performed to compare relative frequencies, and the Mann-Whitney *U*-test to test for significant differences in the distributions between unpaired data sets. In experiments in which multiple groups were compared (e.g., **Figure 4**), we applied the Bonferroni correction of alpha to control the familywise error rate at 0.05 (alpha is given in legends). The numbers of cricket fights for each experiment and test group are indicated in the figures.

## Results

### Immediate response to antennal stimulation (AS)

**Figure 1:**
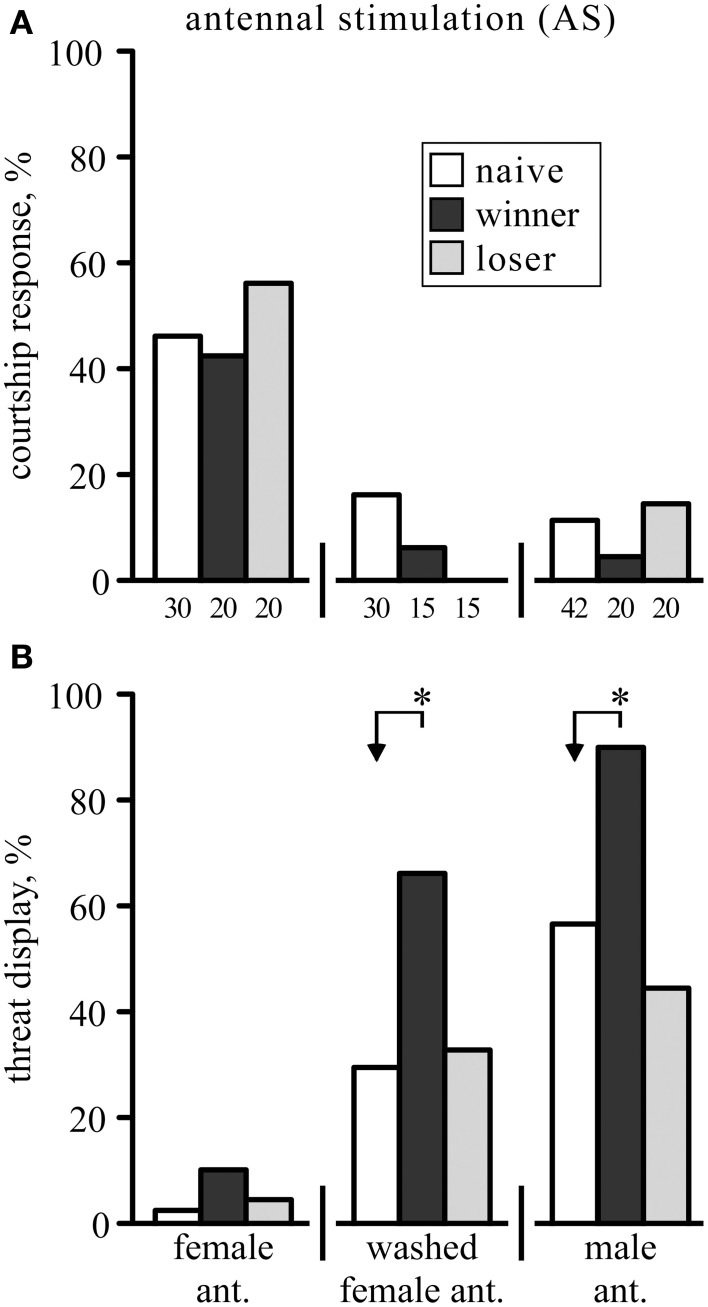
**The behavioral responses of male crickets to antennal stimulation (AS)**. The animals showed either: **(A)** courtship related responses (advertising calling song and/or courtship song), **(B)** the aggressive threat display (mandible spreading), or no response (not shown). The frequencies of these responses (%) are shown for crickets that were either socially naive (white bars), winners (black bar) or losers (gray bar), and stimulated (from left to right) with either an unwashed or washed female antenna, or a male antenna. Note that the animals were different for each test group, so that each animal was stimulated only once with the donor antenna. Significant differences between data sets are indicated by asterisks (χ^2^ test, Bonferroni correction to alpha for two comparisons: ^*^p < 0.025; *n* is given below the x-axis in **A**).

The response to stroking a male crickets antenna with another single antenna depended on the sex of its donor. In response to stimulation with a fresh, unwashed female antenna (female-AS) socially naive male crickets tend to generate courtship related singing (46.7%, *n* = 30; Figure 1A), which could be either the advertising calling song (36.7%) or courtship song alone (3.3%) or bouts of both (6.7%). Mandible spreading, a characteristic threat display (TD) in crickets (Judge and Bonanno, 2008) was observed in only one case (3.3%, Figure 1B). Singing occurred significantly less often when the female antenna was washed with n-hexane to remove cuticular pheromonal compounds (17%, χ^2^ = 6.3, *p* = 0.012), and significantly more crickets responded with the TD (30%, *n* = 30, χ^2^ = 7.7, *p* = 0.0055). Finally, AS with a fresh, unwashed male-antenna (male-AS) primarily induced the aggressive TD (57%, *n* = 42, significantly different to AS with washed female antenna: χ^2^ = 5.1773, *p* = 0.023). In these cases the TD was frequently combined with short bouts of the aggressive rival song (43%), but never together with the calling or courtship song, either of which did however occur alone in 12% of the socially naive males.

Since winning promotes cricket aggression and losing suppresses it (Rillich and Stevenson, 2011; Stevenson and Rillich, 2013) we also tested how these social experiences influence the animal's responsiveness to AS. Winners of a previous fight did not respond differently to female-AS (43% courting, 10% TD, *n* = 20), but showed the TD significantly more frequently than naive males when stimulated with a washed female antenna (67% *vs*. 30%, χ^2^ = 5.6, *p* = 0.018) or with an unwashed male antenna (90% *vs*. 57%, χ^2^ = 6.7, *p* = 0.0094; Figure 1B). Contrasting this, the behavioral responses to AS were not different in losers compared to socially naïve crickets (χ^2^, *p* > 0.05) irrespective of whether stimulated with a female (57% courting, 5% TD, *n* = 20), washed female (0% courting, 33% TD, *n* = 15) or male antenna (15% courting, 45% TD, *n* = 20).

**Figure 2:**
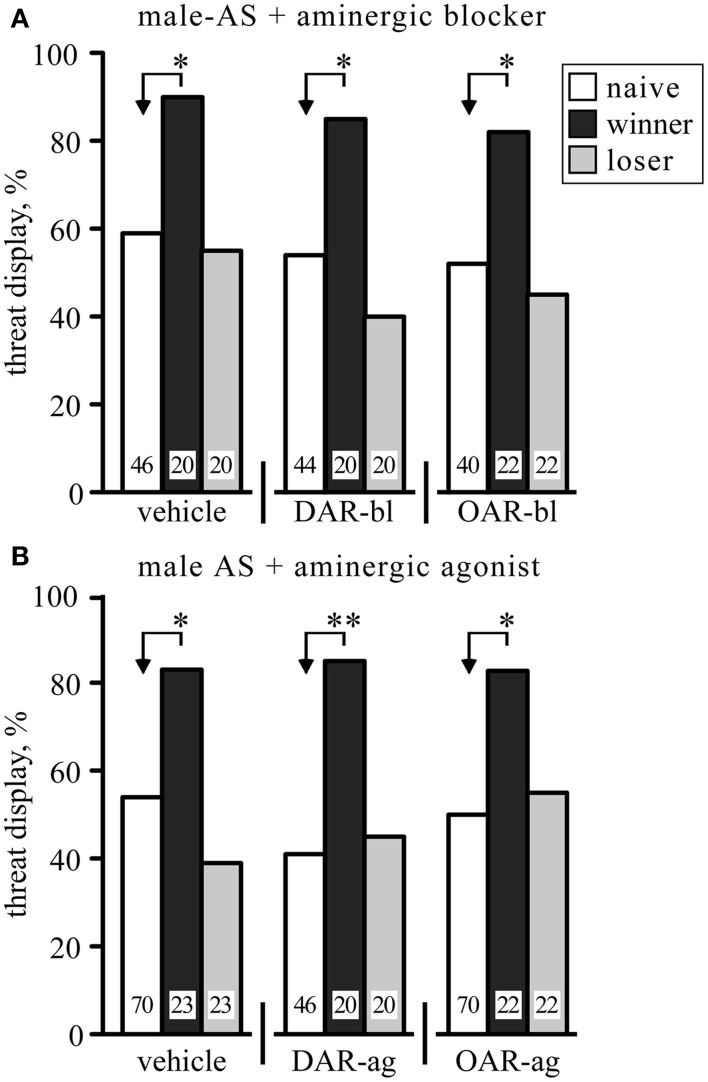
**Influence of aminergic blockers (A) and agonists (B) on the frequency of threat display exhibited by male crickets in response to stimulation with a male antenna (male-AS)**. Data are shown for crickets that were either socially naive (white bars), winners (black bar), or losers (gray bar). Note that the animals were different for each test group, so that each animal was stimulated only once with the donor antenna. In **(A)** the crickets received, from left to right, vehicle (1% DMSO in saline), the DA receptor blocker fluphenazine (DAR-bl) or the OA receptor blocker epinastine (OAR-bl). In **(B)** the crickets received, from left to right, vehicle, the DA receptor agonist homovanillyl alcohol (DAR-ag) or the OA receptor agonist chlordimeform (OAR-ag). Significant differences between data sets are indicated by asterisks [χ^2^ test, Bonferroni correction to alpha for two comparisons: ^*^p < 0.025; ^**^p < 0.005; *n* is given above the x-axis in **(A)**]. All data are given in Table 1.

Since the expression of aggression is promoted by OA (Stevenson and Rillich, 2012) and DA (Rillich and Stevenson, 2014), we tested whether selective OAR and DAR antagonists influence the responses of socially naïve crickets to male-AS. However, compared to vehicle (1% DMSO in saline), neither the OAR-blocker epinastine nor the DAR-blocker fluphenazine significantly influenced the efficacy of male-AS to elicit the aggressive TD (relative frequencies not significantly different, χ^2^, *p* > 0.05; Figure 2A, numeric data in Table 1). Similarly, the frequency of the TD exhibited in response to male-AS was also not changed by the OAR-agonist chlordimeform (CDM) or the DAR-agonist HVA compared to vehicle (χ^2^, *p* > 0.05; Figure 2B, Table 1). Furthermore, although the promoting effect of winning on aggression is mediated by OA (Rillich and Stevenson, 2011), the promoting effect of winning on the response to male-AS was not affected by treatment with the selected OAR- and DAR-blockers or agonists (Figure 2, Table 1). For example, the winners still responded significantly more frequently with the TD compared to socially naive crickets when treated with the OAR-blocker epinastine (naïve 52.5%, winners 82%, χ^2^ = 5.29, *p* = 0.021).

**Table 1:** **Frequency of threat display (%) exhibited by male crickets in response to stimulation with a male antenna and sample number (*n*) for data depicted in Figure 2**.

	**Threat display frequency %, (*n*)**		
	**Naive**	**Winners**	**Losers**
Vehicle	59 (46)	90 (20)	55 (20)
DAR-blocker	55 (44)	85 (20)	40 (20)
OAR- blocker	53 (40)	82 (22)	46 (22)
Vehicle	54 (70)	83 (23)	39 (23)
DAR-agonist	41 (46)	85 (20)	45 (20)
OAR-agonist	50 (70)	82 (22)	55 (22)

*The crickets were either socially naive, winners or losers and treated with the DAR-blocker fluphenazine, the OAR-blocker epinastine, the DAR-agonist homovanillyl alcohol or the OAR-agonist chlordimeform, and compared to vehicle (1% DMSO, separate controls for blockers and agonists)*.

**Figure 3:**
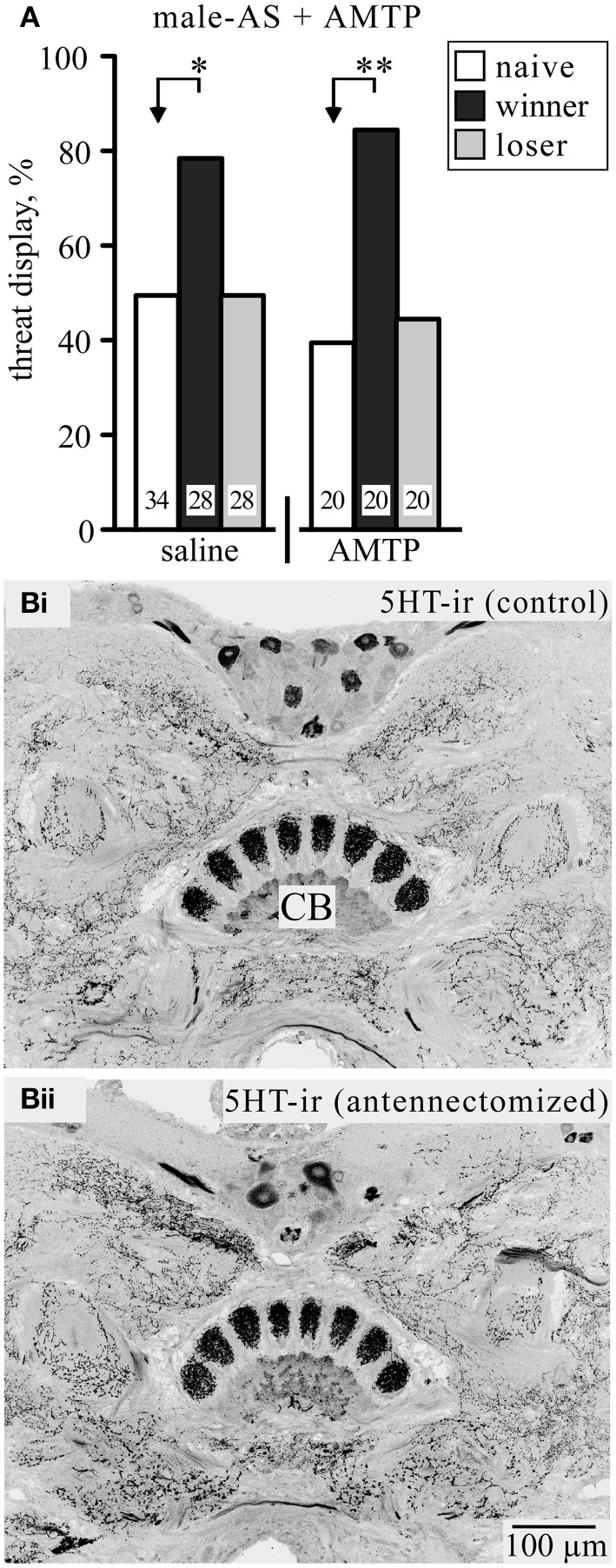
**(A)** The competitive serotonin (5HT) synthesis inhibitor alpha-methyl-tryptophan (AMTP) has no effect on the frequency of threat display in response to male-AS. Data are given for socially naive crickets (white bars), winners (black bar), and losers (gray bar). Significant differences between data sets are indicated by asterisks [χ^2^ test, Bonferroni correction to alpha for two comparisons: ^*^p < 0.025; ^**^p < 0.005; *n* is given above the x-axis in **(A)**]. **(B)** Frontal sections of a male cricket's brain showing 5HT-like immunoreactive (5HT-ir) staining of the central body (CB): (**Bi)** from a control cricket, (**Bii)** a corresponding section from a cricket 7 days after abating both antennae.

It has been shown that a precursor for the amine serotonin (5-hydroxytryptamine, 5HT) prolongs the duration of the TD during actual fighting (Dyakonova and Krushinsky, 2013). We therefore tested the effect of the 5HT synthesis inhibitor alpha-methyl-tryptophan (AMTP) on the response to male-AS (Figure 3A). At a dosage that effectively depletes 5HT from the cricket brain (cf. Sloley and Orikasa, 1988; Stevenson et al., 2000) AMTP-treated socially naive crickets did not respond differently to vehicle treated crickets following male-AS (TD frequency, AMTP: 40%, *n* = 20; saline: 50%, *n* = 34, χ^2^ = 0.5, *p* = 0.47). Furthermore, 5HT-depleted winners still responded significantly more frequently with the TD (85%, *n* = 20) compared to socially naive crickets (χ^2^ = 8.64, *p* = 0.0033) and losers (45%, *n* = 20, χ^2^ = 7.03, *p* = 0.008). Another issue with 5HT is the claim that the tendency of male crickets to court rather than fight other males after antennectomy (Hofmann and Schildberger, 2001; Sakura and Aonuma, 2013) results directly from depletion of 5HT, particularly in the central body of the brain (Murakami and Itoh, 2003). In contrast to Murakami and Itoh (2003), however, we found no effect of antennectomy on the intensity of 5HT-like immunoreactivity in the cricket central body and surrounding brain neuropils (*n* = 6, e.g., Figure 3B).

### Effect of prior antennal stimulation on fighting behavior

**Figure 4:**
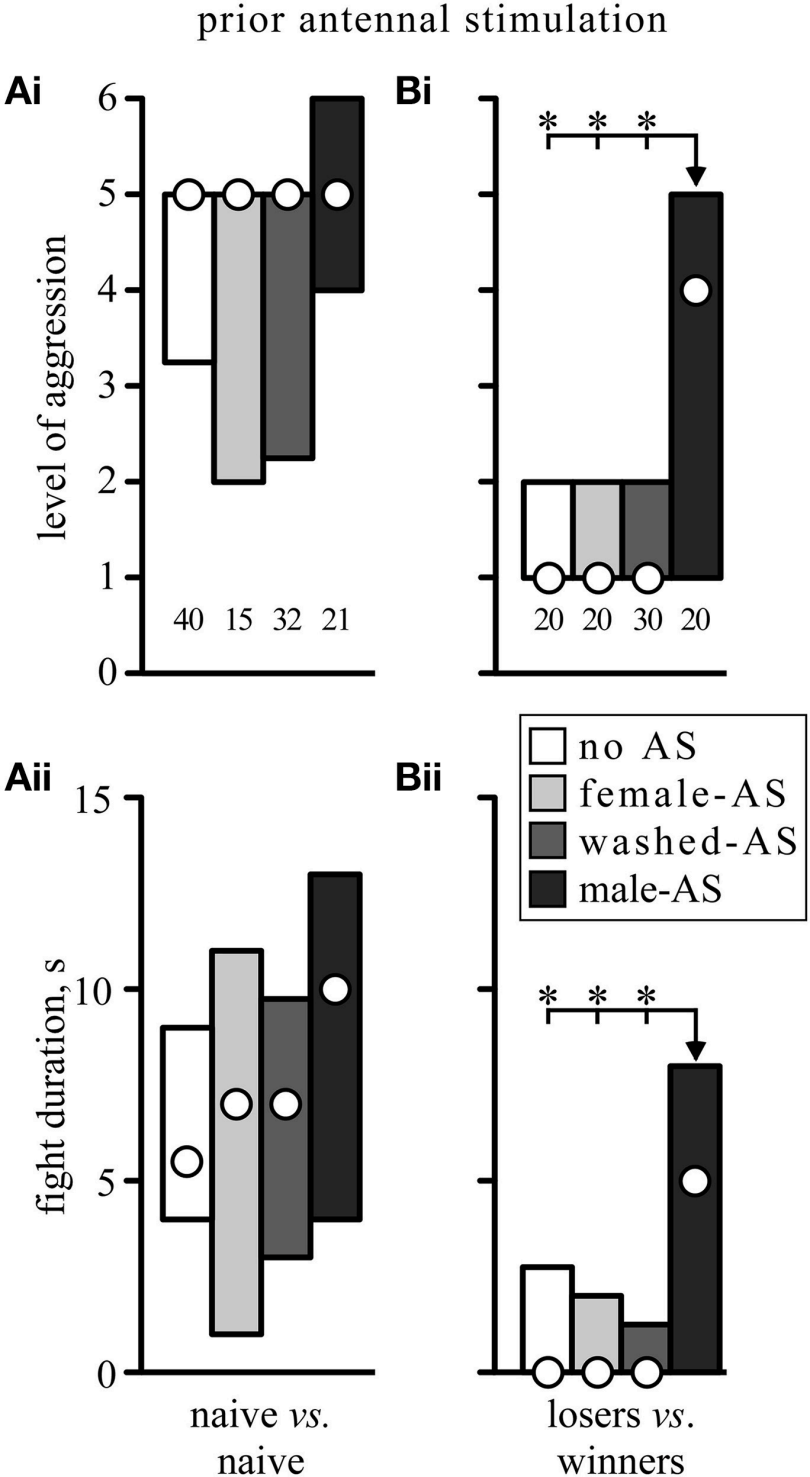
**Effect of prior antennal stimulation on fighting behavior. (A)** Gives the escalation level **(Ai)** and duration **(Aii)** of fights between socially naive crickets, and **(B)** for fights between winners and losers 15 min after an initial fight [different animals to those in **(A)**, circles: median, boxes: interquartile range, *n* is given above the x-axis in **(Ai)** and **(Bi)**]. Five minutes prior to each fight the animals received either no antennal stimulation (white bars) or stimulation with an unwashed female antenna (light gray bars), a washed female antenna (dark gray bars), or a male antenna (black bars). Asterisks indicate significant differences between data sets (*U*-test: ^*^p < 0.0167 to accommodate Bonferroni correction to alpha for 3 comparisons).

In addition to initiating an immediate behavioral response, AS also had a priming effect on aggressive behavior in that it enhanced its subsequent expression, but this was only statistically significant following male-AS in subordinates (losers) which are normally non-aggressive. As shown in Figure 4A, socially naïve crickets that received no prior AS typically escalated to the level of mandible engagement (median level 5, IQR 3.25–5, *n* = 40, Figure 4Ai) in fights that lasted between 4 and 9 s (IQR, median duration 5.5 s, Figure 4Aii). In these normally aggressive socially naive crickets, AS with a female antenna, a washed antenna or a male antenna had no statistically significant effect on the escalation level or duration of the fights, despite a trend toward higher aggression levels and longer fights after male-AS. Contrasting this, prior AS had a profound effect on the aggressiveness of losers matched against winners. As illustrated in Figure 4B, losers normally retreated from the previous winners 15 min after defeat (median level 1, IQR 1–2, *n* = 20), and this was not significantly changed by prior stimulation of the loser with a female or washed antenna (female-AS: median level 1, IQR 1–2, *n* = 20, washed-AS: median level 1, IQR 1–2, *n* = 30). However, 5 min after male-AS, many of the losers exhibited aggression toward the previous winners (median level 4, IQR 1–5, *n* = 20, *U*-tests compared to no AS: *p*-level = 0.0069, *p*-duration = 0.0038). In fact, more than 50% of all losers exhibited the aggressive TD toward their previous victors and 30% even escalated to physical engagements. This priming effect on fighting behavior results from AS, rather than the TD evoked by it, since priming was also evident in crickets that did not respond to prior AS with TD (7 of 11 non-responders compared to 7 of 9 responders).

**Figure 5:**
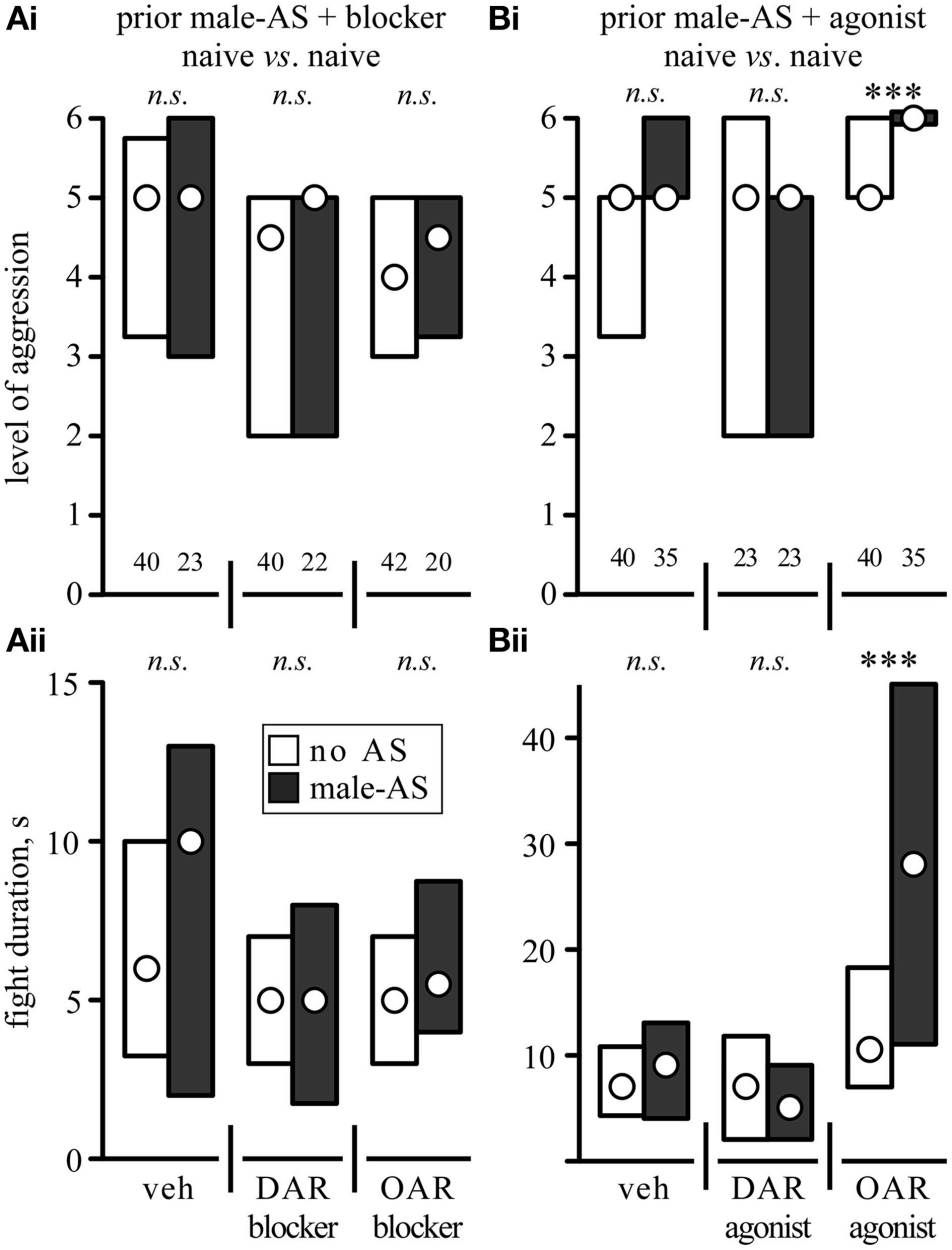
**Influence of (A) aminergic blockers and (B) agonists on the enhancing effect of prior male-AS on fights between socially naive male crickets [(Ai,Bi) escalation level, (Bi,Bii) fight duration; circles: median, boxes: interquartile range, *n* is given above the x-axis in (Ai,Bi)]**. The animals received either no AS (white bars) or AS with a male antenna 5 min prior to each fight (black bars). The crickets were also treated, from left to right, with either: **(A)** vehicle (veh, 1% DMSO in saline), the DAR-blocker fluphenazine or the OAR-blocker epinastine. **(B)** Vehicle (veh, 1% DMSO in saline), the DAR-agonist homovanillyl alcohol or the OAR-agonist chlordimeform. Asterisks indicate statistically significant differences between data sets (*U*-test: ^***^p < 0.001, *n.s*., not significant). All data are given in Table 2.

We next investigated whether or not the priming effect of male-AS on subsequent aggression was influenced by amine-receptor antagonists or agonists (Figure 5, Table 2). We first evaluated fights between naive crickets, where prior-AS had little if any effect on subsequent aggression. Firstly, and in line with the data shown in Figure 4, fights between socially naive male crickets treated with vehicle were not affected by prior male-AS (Figure 5A, no AS: median level 5, IQR: 3.25–5.75, median duration 6 s, IQR: 3.25–10, *n* = 40; male-AS: median level 5, IQR: 3–6, median duration 10 s, IQR: 2–13, *n* = 23). Secondly, and confirming our earlier studies (Rillich and Stevenson, 2014), the aggression of socially naive crickets without male-AS was reduced by both the DAR-blocker fluphenazine (*U*-tests compared to vehicle: *p*-level = 0.027*, p*-duration = 0.067, Figure 5A) and the OAR-blocker epinastine (*U*-tests compared to vehicle: *p*-level = 0.036, *p*-duration = 0.087, Figure 5A), but this was not significantly changed further by prior male-AS (*U*-tests: DAR-blocker *p*-level = 0.698, *p*-duration = 0.836; OAR-blocker *p*-level = 0.399, *p*-duration = 0.288, Figure 5A). Thirdly, the DAR-agonist HVA had no effect on the aggression of socially naive crickets (*U*-tests compared to vehicle: *p*-level = 0.46*, p*-duration = 0.34, Figure 5B), and HVA-treated crickets did not fight significantly different after prior male-AS (*U*-tests compared to no As + HVA: *p*-level = 0.489, *p*-duration = 0.42, Figure 5B). Finally, and in contrast to HVA, the OAR-agonist CDM increased aggression of socially naive crickets that did not receive prior male-AS (*U*-tests compared to vehicle: *p*-level = 0.027, *p*-duration = 0.0175, Figure 5B). This aggression promoting effect of CDM was far more dramatic after male-AS, practically all fights in this test group escalated to the highest level (median 6, IQR: 6-6, *n* = 35) and lasted from 11 to 45 s (IQR, median 28 s, *U*-tests compared to no AS + CDM: *p*-duration < 0.001, *p*-level < 0.001, Figure 5B). Thus, in the presence of the OAR-agonist CDM, prior male-AS has a pronounced priming effect on the expression of aggression in socially naive crickets.

**Table 2:** **The level and duration of fights (median, IQR and *n*) between socially naïve crickets that received either no antennal stimulation (no AS) or stimulation with a male antenna 5 min before fighting (male-AS)**.

**Naive *vs*. naive**	**Fight level** **median, IQR (*n*)**			**Fight duration, s median, IQR**		
	**No AS**	**Male-AS**	***p*-value**	**No AS**	**Male-AS**	***p*-value**
Vehicle	5, 3.25–5.75 (40)	5, 3–6 (23)	0.346	6, 3.25–10	10, 2–13	0.457
DAR-blocker	4.5, 2–5 (40)	5, 2–5 (22)	0.698	5, 3–7	5, 1.75–8	0.836
OAR-blocker	4, 3–5 (42)	4.5, 3.25–5 (20)	0.399	5, 3–7	5.5, 4–8.75	0.288
Vehicle	5, 3.25–5 (40)	5, 5–6 (35)	0.0923	7, 4.25–10.75	9, 4–13	0.421
DAR-agonist	5, 2–6 (23)	5, 2–5 (23)	0.489	7, 2–11.75	5, 2–9	0.42
OAR-agonist	5, 5–6 (40)	6, 6–6 (35)	**<0.001**	10.5, 7–18.25	28, 11–45	**<0.001**

*The crickets were treated with either: the DAR-blocker fluphenazine, the OAR-blocker epinastine, the DAR-agonist homovanillyl alcohol or the OAR-agonist chlordimeform, or vehicle (1% DMSO, separate controls for blockers and agonists). The p-values from U-tests comparing groups that received no AS and male-AS are given for each treatment (statistically significant values in bold face). The data are depicted in Figure 5*.

**Figure 6:**
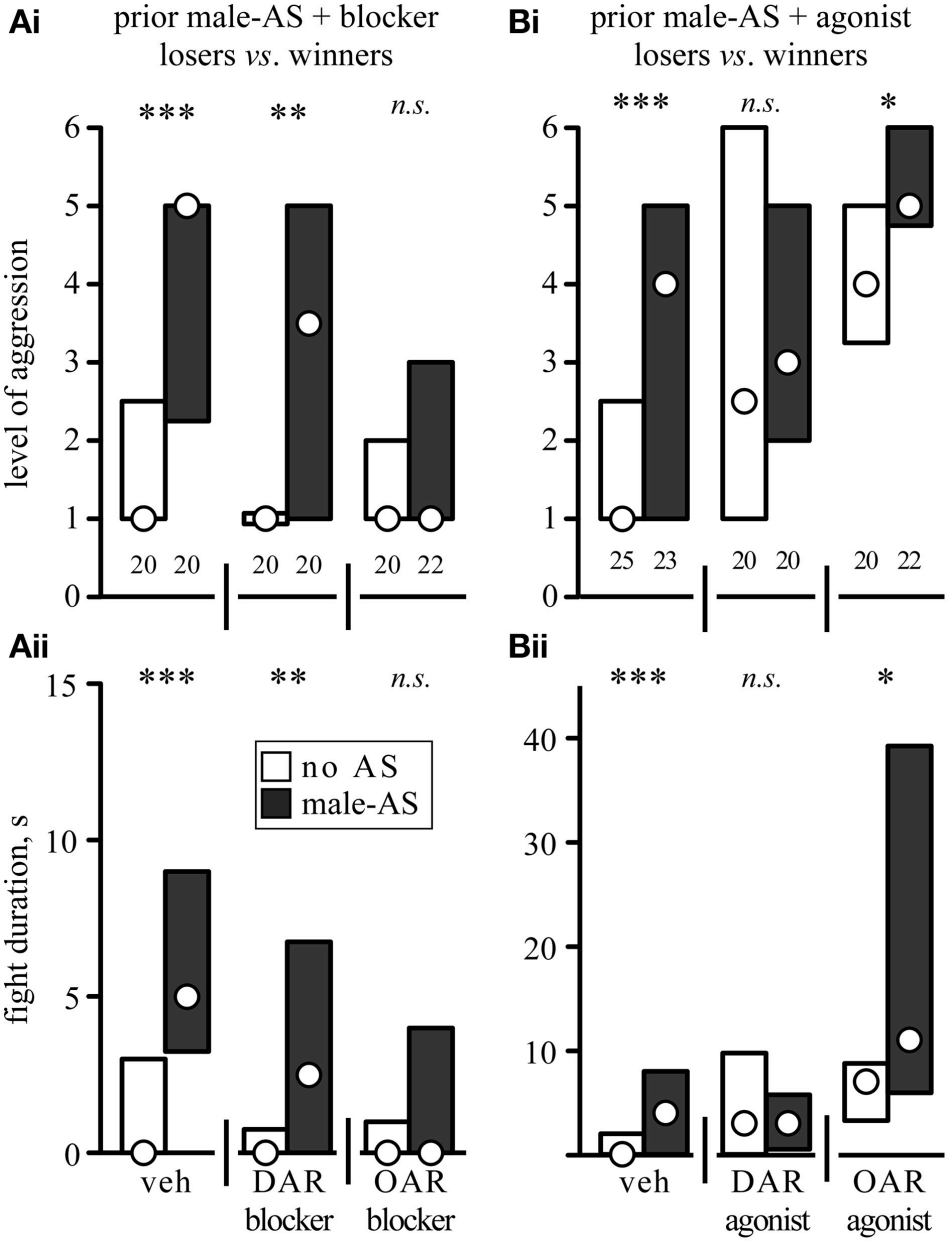
**Influence of (A) aminergic blockers and (B) agonists on the enhancing effect of prior male-AS on fights between losers against winners matched 15 min after a previous fight (labels as in Figure 5, but for different animals)**. Asterisks indicate statistically significant differences between data sets (*U*-test: ^*^p < 0.05, ^**^p < 0.01, ^***^p < 0.001, *n.s*., not significant). All data are given in Table 3.

The effects of the aminergic drugs were more pronounced in interactions of losers matched against their previous victors (Figure 6, Table 3). In line with the data for untreated losers (Figure 4B), vehicle treated losers retreated from winners (median level 1, IQR: 1–1.75, median duration 0 s, IQR: 0–3, *n* = 20), but became significantly more aggressive after male-AS (median level 5, IQR: 2.25–5, median duration 5 s, IQR: 3.25–9, *n* = 20, *U*-tests: *p*-duration < 0.001, *p*-level < 0.001, Figure 6A). This enhancing effect of male-AS was also still evident in losers treated with the DAR-blocker fluphenazine (*U*-tests compared no-AS: *p*-level = 0.0033, *p*-duration = 0.0058, Figure 6A), but not in losers treated with the OAR-blocker epinastine (male-AS: median level 1, IQR 1–3, median duration 0 s, IQR 0–4, *n* = 22, *U*-test compared to no AS: *p*-level = 0.912, *p*-duration = 0.832). As found in a previous study (Rillich and Stevenson, 2014) the DAR-agonists HVA and the OAR-agonist CDM each increased the expression of aggression in losers matched against winners, even without prior male-AS (*U*-tests compared to vehicle, HVA: *p*-level = 0.0126, *p*-duration = 0.0134, CDM: *p*-level < 0.001, *p*-duration < 0.001, Figure 6B). We then tested how these agonists influenced the priming effect of prior male-AS. While the contests between losers and winners treated with the DAR-agonist HVA were not significantly affected by prior male-AS (*U*-tests: *p*-level = 0.832, *p*-duration = 0.97), the promoting effect of the OAR-agonist CDM on loser aggression was significantly enhanced by prior male-AS (*U*-tests compared to no AS: *p*-level = 0.023, *p*-duration = 0.04). Hence the priming effect of male-AS on aggression is mediated by OA rather than DA.

**Table 3:** **The level and duration of fights (median, IQR and *n*) between losers matched against winners**.

**Losers *vs*. winners**	**Fight level median, IQR (*n*)**			**Fight duration, s median, IQR**		
	**No AS**	**Male-AS**	***p*-value**	**No AS**	**Male-AS**	***p*-value**
Vehicle	1, 1–1.75 (20)	5, 2.25–5 (20)	**<0.001**	0, 0–3	5, 3.25–9	**<0.001**
DAR-blocker	1, 1–1 (20)	3.5, 1–5 (20)	**0.0033**	0, 0–0.75	2.5, 0–6.75	**0.0058**
OAR-blocker	1, 1–2 (20)	1, 1–3 (22)	0.912	0, 0–1	0, 0–4	0.832
Vehicle	1, 1–2.5 (25)	4, 1–5 (23)	**<0.001**	0, 0–2	4, 0–8	**<0.001**
DAR-agonist	4, 3.25–5 (20)	3, 2–5 (20)	0.8354	3, 0–9.75	3, 1–5.75	0.97
OAR-agonist	2.5, 1–6 (20)	5, 4.75–6 (22)	**0.023**	7, 3.25–8.75	11, 6–39.25	**0.04**

*The losers received either no antennal stimulation (no AS) or stimulation with a male antennae 5 min before fighting (male-AS). The crickets were treated with either: the DAR-blocker fluphenazine, the OAR-blocker epinastine, the DAR-agonist homovanillyl alcohol or the OAR-agonist chlordimeform, or vehicle (1% DMSO, separate controls for blockers and agonists). The p-values from U-tests comparing groups that received no AS and male-AS are given for each treatment (statistically significant values in bold face). The data are depicted in Figure 6*.

## Discussion

Antennal fencing between conspecific crickets is a key-releasing stimulus for evoking courtship between sexes and aggression between males and depends on both the mechanical impact and pheromonal signature of the antennae (Rence and Loher, 1977; Hofmann and Schildberger, 2001; Nagamoto et al., 2005; Iwasaki and Katagiri, 2008; Sakura and Aonuma, 2013). Confirming earlier studies (Rence and Loher, 1977), AS with a female antenna evokes courtship behavior in male crickets, while male-AS evokes mandible spreading, a characteristic aggressive threat display that follows antennal fencing in escalating fighting behavior (Hofmann and Stevenson, 2000). The net effect of AS on aggression is thus notably different to other agonistic signals experienced during fighting, such as the opponent's mandible threat display, which act to suppress the expression of aggression (Rillich et al., 2007), via a mechanism involving the gaseous neuromodulator nitric oxide (Stevenson and Rillich, 2015).

Contrary to some earlier studies (Rence and Loher, 1977; Nagamoto et al., 2005; Iwasaki and Katagiri, 2008) we found that female antennae, that were washed to remove cuticular pheromonal compounds, were almost equally effective as male-AS in initiating aggression (Figure 1). Our data thus illustrate that mechanical stimulation alone, as claimed by Alexander (1961), is sufficient to initiate aggression in crickets, although it need not be entirely necessary (Sakura and Aonuma, 2013) since male odors (Iwasaki and Katagiri, 2008) and cricket song (Rillich et al., 2009) can also elicit an aggressive response.

In the present study, we found that the response to male-AS depends on social status (Figures 1, 2). Winners of a previous fight were far more likely to respond with the aggressive threat display than socially naive crickets. This could occur when crickets that are inherently more responsive to AS win more often, and hence become overrepresented in the winner group. We can rule this out, however, because responders in fact did not win more often against non-responders (54.5% wins, *n* = 33; not statistically different to 50%, data not shown). With respect to losers, these were equally as responsive to male-AS as socially naive crickets, even though they behave submissively toward other males (Stevenson and Rillich, 2013). Hence, losers are still potentially aggressive, and not necessarily motivationally depressed (see also Rillich et al., 2007; Simpson and Stevenson, 2014). We conclude that the experience of winning in crickets increases the efficacy of the natural releasing stimulus to initiate aggressive behavior, as is known in vertebrates (Hsu et al., 2006).

Although OA promotes the expression of aggression in insects (Bubak et al., 2014; Simpson and Stevenson, 2014) and AS excites octopaminergic neurons (Duch et al., 1999), we were surprised to find that octopaminergic drugs failed to influence the efficacy with which AS elicited an aggressive response (Figure 2). Similarly, while the experience of winning enhances the expression of aggression *via* the action of OA (Rillich and Stevenson, 2011), the promoting effect of winning on the responsiveness to male-AS was not blocked by the selective OAR-antagonist epinastine, nor promoted by the OAR-agonist CDM (Figure 2) at concentrations that profoundly affect fighting behavior (Figures 5, 6). We conclude that OA is not essential for initiating aggression and is not responsible for increasing the efficacy of the natural releasing stimulus after winning. This is in line with our finding that crickets lacking OA and DA still express aggressive behavior, albeit at a lower level (Stevenson et al., 2000). In this respect, the initiation of aggression differs to insect flight, for which OA increases the probability of initiation in locusts (Stevenson and Kutsch, 1988) and fruit flies (Brembs et al., 2007) by promoting cholinergic neurotransmission (Buhl et al., 2008; Rillich et al., 2013).

Although DA, like OA, increases general responsiveness to sensory stimulation (Van Swinderen and Andretic, 2011) and the expression of aggression in subordinate crickets (Rillich and Stevenson, 2014), we found that established DAR-antagonists and -agonists had no effect on the aggressive response to male-AS. The amine serotonin (5HT) is also unlikely to be involved in this effect. Although crickets treated with a 5HT precursor exhibit the TD longer during actual fighting (Dyakonova and Krushinsky, 2013), treatment with the synthesis inhibitor AMTP, which effectively depletes 5HT from the brain (Stevenson et al., 2000), did not reduce the effectiveness of male-AS to elicit the aggressive threat response, irrespective of social status (Figure 3A). Furthermore, the tendency of male crickets to court rather than fight other males after antennectomy (Hofmann and Schildberger, 2001) is unlikely to result from loss of 5HT following this operation as claimed by Murakami and Itoh (2003). Contrary to these authors, we found that antennectomy had no effect on the intensity of 5HT-like immunoreactivity in the cricket brain (Figure 3B). Although we cannot fully rule out with this method that antennectomy slightly reduces 5HT levels, our depletion experiments clearly show that this would not change the efficacy of AS as an aggression releasing stimulus. Hence, the transmitter system that ultimately initiates aggression and underlies the enhancing effect of winning on the immediate response to AS still remains to be identified.

In addition to initiating the aggressive threat response, male-AS also led to enhanced aggressiveness of the crickets in subsequently staged fights (Figure 4), which confirms the effectiveness of corresponding procedures employed by Chinese aficionados of cricket fighting (Suga, 2006). Although not statistically significant in socially naive crickets, which are highly aggressive by default (cf. Stevenson and Rillich, 2013), prior male-AS had a dramatic priming effect on normally submissive losers, which frequently engaged and fought their previous victors instead of retreating (Figure 4). While the efficacy of AS to actually initiate aggression was not influenced by OA drugs (Figure 2), the priming effect of AS on subsequent fighting behavior was (Figures 5, 6). Firstly, in naive crickets, where prior-AS had little if any effect, male-AS in combination with the OAR-agonist CDM, but not the DAR-agonist HVA, led to a pronounced increase in aggression: all treated crickets escalated to the highest level (6) and fought up to four times longer than usual. Secondly, the priming effect on losers was selectively enhanced by CDM, but not HVA, and effectively blocked by the OAR-antagonist epinastine, but not by the DAR-antagonist fluphenazine. We conclude that male-AS promotes subsequent fighting behavior *via* the action of OA, once it has been initiated.

Notably, prior stimulation with a washed antenna, which proved equally effective as male-AS in eliciting the threat response, did not promote subsequent fighting behavior, indicating that the priming effect also depends on male pheromones (Figure 4). Supporting this suggestion, OA levels in the haemolymph increase after male-AS, but not after mechanical stimulation alone (Adamo et al., 1995). Furthermore, in the fruit fly, a specific subset of gustatory receptors, that are sensitive to male contact pheromones and play a critical role in male social behaviors, appear to promote aggression *via* activation of octopaminergic neurons (Andrews et al., 2014).

**Figure 7:**
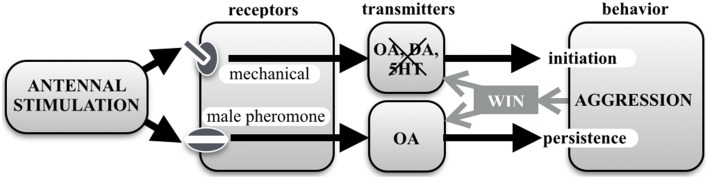
**Chart summarizing this study's results**. Aggressive behavior in crickets is initiated by mechanical antennal stimulation and involves an unknown transmitter system that is neither octopamine (OA), dopamine (DA) or serotonin (5HT). At the same time, contact with a male antenna, and hence contact pheromones, promotes persistence of aggressive behavior (escalation level and fight duration) *via* the action of OA. OA also mediates the promoting effect of winning and experiences such as physical exertion and resource possession, on aggressive persistence (review: Stevenson and Rillich, 2012). Interestingly, winning also enhances the efficacy of mechanical stimulation to initiate aggression, but *via* a different mechanism that does not involve OA, DA or 5HT.

Taken together, our current results, summarized in Figure 7, shed new light on the role of OA in the control of aggression. We conclude that aggressive behavior in crickets is initiated by mechanical AS and the action of an unidentified neurotransmitter, and while this key release system operates more effectively after scoring a win in social conflict, it does so without the involvement of OA, DA, or 5HT. OA seems rather to act as a modulator over a longer time scale to promote the persistence of aggressive behavior once initiated. We have previously shown that the experiences of physical exertion (Stevenson et al., 2005), winning (Rillich and Stevenson, 2011) and resource possession (Rillich et al., 2011), all act to increase a cricket's tendency to persist in aggression *via* the action of OA. Similarly, in this study, experiencing prior antennal contact with a male conspecific, promotes subsequent fighting behavior via activation of the octopaminergic system, most likely by afferents sensitive to male contact pheromone as in fruit flies (Andrews et al., 2014). Our experiments thus again reveal crickets as an ideal model system for teasing apart the specific functions of biogenic amines in controlling aggression. It remains to be established whether the role of amines in other animal model systems for aggression is also restricted to a modulatory, rather than a releasing, function.

## Author contributions

Conceived and designed the experiments: JR PS. Performed the experiments: JR PS. Analyzed the data: JR PS. Contributed reagents/ materials/ analysis tools: PS JR. Wrote the paper: PS JR.

### Conflict of interest statement

The authors declare that the research was conducted in the absence of any commercial or financial relationships that could be construed as a potential conflict of interest.
